# FMRI connectivity analysis of acupuncture effects on an amygdala-associated brain network

**DOI:** 10.1186/1744-8069-4-55

**Published:** 2008-11-13

**Authors:** Wei Qin, Jie Tian, Lijun Bai, Xiaohong Pan, Lin Yang, Peng Chen, Jianping Dai, Lin Ai, Baixiao Zhao, Qiyong Gong, Wei Wang, Karen M von Deneen, Yijun Liu

**Affiliations:** 1 Medical Image Processing Group, Institute of Automation, Chinese Academy of Sciences, Beijing, 100080, PR China; 2 Life Science Research Center, School of Electronic Engineering, Xidian University, Xi’an, Shaanxi 710071, PR China; 3 Department of Radiology, Beijing Tiantan Hospital, Capital University of Medical Sciences, Beijing, 100050, PR China; 4 Department of Anatomy and Embryology, Capital Medical University, Beijing, 100069, PR China; 5 Beijing University of Chinese Medicine, Beijing, 100029, PR China; 6 Department of Psychology, School of Education Science, East China Normal University, Shanghai, 200241, PR China; 7 Huaxi MR Research Center, Department of Radiology, Medical Imaging Center, West China Hospital of Sichuan University, Chengdu, Sichuan 610054, PR China; 8 The Fourth Military Medical University, Xi’an, Shaanxi 710038, PR China; 9 Departments of Psychiatry and Neuroscience, McKnight Brain Institute, University of Florida, Gainesville, FL 32610, USA

## Abstract

**Background:**

Recently, increasing evidence has indicated that the primary acupuncture effects are mediated by the central nervous system. However, specific brain networks underpinning these effects remain unclear.

**Results:**

In the present study using fMRI, we employed a within-condition interregional covariance analysis method to investigate functional connectivity of brain networks involved in acupuncture. The fMRI experiment was performed before, during and after acupuncture manipulations on healthy volunteers at an acupuncture point, which was previously implicated in a neural pathway for pain modulation. We first identified significant fMRI signal changes during acupuncture stimulation in the left amygdala, which was subsequently selected as a functional reference for connectivity analyses. Our results have demonstrated that there is a brain network associated with the amygdala during a resting condition. This network encompasses the brain structures that are implicated in both pain sensation and pain modulation. We also found that such a pain-related network could be modulated by both verum acupuncture and sham acupuncture. Furthermore, compared with a sham acupuncture, the verum acupuncture induced a higher level of correlations among the amygdala-associated network.

**Conclusion:**

Our findings indicate that acupuncture may change this amygdala-specific brain network into a functional state that underlies pain perception and pain modulation.

## Background

Acupuncture is one of the most important therapeutic modalities in traditional Chinese medicine (TCM). It utilizes fine needles that may pierce through specific anatomical points (named '*acupoints'*) so that certain healing effects are produced. In clinical practice, sensation induced by needling at acupuncture points is asserted as '*deqi*', and the resulting effects of acupuncture stimulation have been ascribed to treatment of various diseases [1]. While acupuncture has gained much popularity in the Western medical community, the underlying mechanisms remain mostly unknown.

Previous human neuroimaging studies have shown that acupuncture stimulation activates extensive brain regions, including the primary somatosensory cortex (SI), secondary somatosensory cortex (SII), anterior cingulate cortex (ACC), insular cortex, prefrontal cortex (PFC), amygdala, hippocampus, periaquaductal gray (PAG) and hypothalamus [2-7]. These distributed brain regions are associated closely with a wider pain matrix for modulating sensations and affective pain perception. Some of these brain regions are also implicated in endogenous anti-nociceptive signaling. Using functional magnetic resonance imaging (fMRI), Wager et al [8] demonstrated that expectancy might modulate the pain matrix, along with a considerable overlap among the brain areas in response to placebo and expectation. A recent PET study by Pariente et al [9] has identified different areas of activations induced by both the expectation of acupuncture and actual acupuncture. These findings suggest that actual acupuncture may not only activate a brain network associated with expectation and placebo response but also the brain regions implicated in the actual effect of acupuncture analgesia.

Acupuncture, however, is a complex intervention that is intimately intertwined with placebo, patients, and practitioners. We thereby hypothesized that acupuncture may affect this pain matrix in both specific and non-specific manners which contribute to its specific therapeutic effects, as well as the effects of expectation for pain relief. We further questioned whether there are interactions among these brain regions activated during acupuncture intervention. We speculate that these brain regions involved in the pain matrix may constitute various networks to mediate both specific and non-specific effects of acupuncture, which can be assessed using fMRI connectivity analysis methods.

Recently, new but promising fMRI connectivity analysis methods have provided insight into the brain networks mediating acupuncture effects. The term fMRI connectivity describes brain regions that are functionally related and interdependently connected [10,11] by detecting the coherence in fMRI signals among these regions during either a behavioral task or a resting state engaging no task. The between-region correlation during a resting state may represent synchronous fluctuations with a high temporal coherence and reflect intrinsic neuronal connections that coordinate activities in the brain, even for those regions in remote locations [12-14]. Most resting state connectivity studies have employed a 'seeding' approach, in which a seed voxel or several voxels are selected as a functional reference, and then the averaged time course of the fMRI signal from the seeding area is cross-correlated with the time course of each voxel over the entire brain to generate connectivity maps (for details see Methods). Such a functional reference is often determined by a region of interest (ROI) in a brain activation study using specific behavioral tasks or external stimuli [14]. The choice of such ROI is therefore vital and should be carefully defined in functional connectivity analysis.

Acupuncture may recruit distributed cortical and subcortical brain networks that are also implicated in both inhibitory and facilitating effects in the pain-modulation system for both sensation and affective pain perception. Accumulating evidence suggests that the amygdala is an important neural substrate of such reciprocal interaction, and one that also appears to play a key role in the modulation of pain behavior and nociceptive processing at different levels of the pain matrix [15-17]. Furthermore, increasing attention has been paid to the acupuncture-induced deactivation of the amygdala in humans using ST36 or LI4 when contrasting acupuncture needle stimulation with non-stimulation baseline [2,3], to further demonstrate this in relation to the analgesic effect of acupuncture. Results of animal studies have demonstrated that the amygdala formation has abundant opiates receptors and participates in both opioid analgesia and acupuncture analgesia [18]. These findings are noteworthy because the amygdala modulation may demonstrate an acupuncture specificity [19]. In the aforementioned analysis, therefore, we selected the amygdala as the seeding ROI to conduct our functional connectivity analysis. Targeting the brain circuits involving the amygdala using fMRI may improve our understanding of neural mechanisms underlying both acupuncture specific and non-specific effects.

Previous fMRI activation studies have been mostly based on a block paradigm design that detects acupuncture effects according to a presumable temporal pattern of brain activation induced by acupuncture administration [2,3,20]. A block design, or model-dependent approach in general, may not be optimal to the study of acupuncture effects. For example, in a model-dependent block design for specific visual or motor tasks, the corresponding visual or motor cortical areas are assumed to be activated almost simultaneously. This approach, however, is not valid in cases when limited or no prior temporal information is available, such as testing the acute effects of a new drug or food intake on the brain [21]. According to the theory of TCM, acupuncture may induce long lasting post-administration effects [22]. Thus, the actual temporal information for acupuncture-induced changes in brain activity remains lacking. In addition, because of a sustained effect, the 'off-state' in the block design may still retain some acupuncture effect, which has not ideally returned to a baseline. Therefore, using several stimulation blocks in a short period of time, investigators may not be able to dissociate the long lasting effects from other confounding changes, such as the effect of needle manipulation during the experiment. In the current study, a new experimental paradigm, namely the non-repeated event-related fMRI (NRER-fMRI) design, was employed for investigating sustained effects after acupuncture administration by using functional connectivity analysis.

## Methods

### Subjects

The experiment was performed on 18 right-handed healthy Chinese college students (9 males and 9 females age of 24.2 ± 2.9 years old). Subjects with a medical history of any neurological or psychiatric diseases were excluded from study. All the participants have given informed consent approved by a local review board for human studies. None of them had previous acupuncture experience or had been exposed to a high magnetic field.

### Experimental protocol

**Figure 1:**
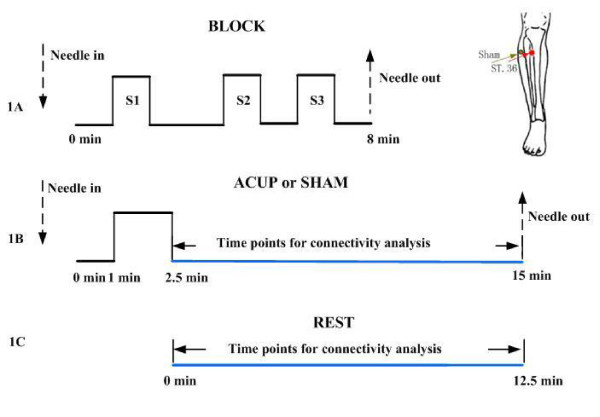
**Experimental design**. A: The design for a conventional block run (BLOCK) with three acupuncture stimulation periods (S1, S2, and S3) lasting for 8 minutes; B: The design for an verum acupuncture (ACUP) run or sham (SHAM) run totally lasting for 15.0 minutes; C: The design for a resting state (REST) run lasting for 12.5 minutes. The images acquired during the time points labeled by blue-color were used for connectivity analyses.

We first used a conventional block design adapted from Hui et al to study brain activation during acupuncture administration [2,3]. In the BLOCK run (Fig. 1A), the participant underwent a conventional block of acupuncture stimulation at ST 36 for 8 minutes with a needle inserted perpendicularly to the skin at a depth of 2–3 cm. One minute later, the needle was manipulated by rotating it clockwise and counterclockwise 60 times per min for 1 minute. Three stimulation blocks S1, S2 and S3 were separated by 2 minutes for S1-S2 or 1 minute for S2-S3 with the needle kept in the point. The scanning continued for 1 minute after S3.

We then employed the new NRER-fMRI design, in which two functional runs: verum acupuncture (ACUP) and sham acupuncture (SHAM) were conducted and only one single stimulation period was given during each of these two runs (Fig. 1B). All participants' eyes were covered so that they could not observe any of the procedures, verum or sham, while they were occurring. The acupoint of ST 36 is located 3 mm lateral and distal to the anterior tubercle of the tibia, which has been examined in various studies and has been proved to have various effects, including efficacy in the treatment of gastric and intestinal diseases in humans and animals. In the verum acupuncture run, an acupuncture needle was inserted at ST 36 from the beginning, and after resting for 1 min, the needle was manipulated for 1.5 minutes; then, the needle remained inserted at the acupoint for another 12.5 minutes. In the sham acupuncture run, the procedure was the same as verum run except that the stimulation was administered at a non-acupoint (2–3 cm apart from ST 36). For both ACUP and SHAM runs, the method for needle manipulation and the depth were all identical to those in the BLOCK run. For a baseline control, a resting state (REST) scan was conducted for 12.5 minutes without any stimulation (Fig. 1C). During the REST run, the participant was asked to remain relaxed without engaging in any mental tasks. All of the subjects affirmed to keep awake during the whole process according to their report after the scanning.

Despite considerable neuroimaging studies on specific effects of acupuncture, the results are controversial and do not demonstrate clear effects of acupuncture over the placebo or controls used in many of previous studies. Some of the drawbacks may be due to poor paradigm design and very limited understanding of the concepts involved in both verum acupuncture and sham. Therefore, the true extent of acupuncture's specific effect cannot be defined without the knowledge of the nature and mechanism of placebo. The choice of placebo or control is vital and needs careful consideration. Our sham control involves the insertion of needles in non-acupoint using the same needling manipulation as in real acupuncture, which is thought to be a classic placebo and well accepted in TCM. In fact, this control has a physiological effect possibly through the mechanisms such as diffuse noxious inhibitory control [23], as well as many of the central neural substrates that are involved in pain [24]. Actual acupuncture given in the guise of this control which has more resembled needling sensation as real acupuncture and stronger blinding to subjects would provide strong support for the existence of acupuncture specific effects.

Because there is a potential long-lasting effect following acupuncture administration [22], a 24-hour interval was taken between the four of above fMRI runs. The presentation sequence of the above four conditions (BLOCK, ACUP, SHAM and REST run) was randomized and balanced throughout the subjects, and every subject performs only one run in each day. And all the subjects completed the four runs. The subjects were informed of acupuncture being administered on the right lower extremity (on ST 36 or sham acupoint), and would feel various sensations, but were blinded to the sequence of the stimulation conditions. The subjects lied down supine inside of the magnet and kept their eyes closed for the entire fMRI run. The stimulation was administered by a balanced 'tonifying and reducing' technique using a sterile disposable 38 gauge stainless steel acupuncture needle, 0.2 mm in diameter and 40 mm in length. The entire acupuncture procedure was conducted by the same experienced and licensed acupuncturist (CP).

### Functional Imaging

Subjects were scanned in a 3.0 Tesla Signa (GE) MR whole body Scanner. A foam pillow and a band (across the forehead) were used to restrict head movement. Functional images were collected in a sagittal orientation parallel to the AC-PC plane with 5 mm slice thickness (no gaps) using a single-shot gradient-recalled echo planar imaging (EPI) sequence. The EPI pulse sequence had the following parameters: TE = 30 ms, TR = 1500 ms, flip angle = 90 degree; matrix size = 64 × 64, FOV 240 × 240 mm^2^, giving an in-plane resolution = 3.75 × 3.75 mm. The scan covered the entire brain including the cerebellum and brainstem. High-resolution structural scans were acquired using 3D MRI sequences with a voxel size of 1 mm^3 ^for anatomical localization.

**Figure 2:**
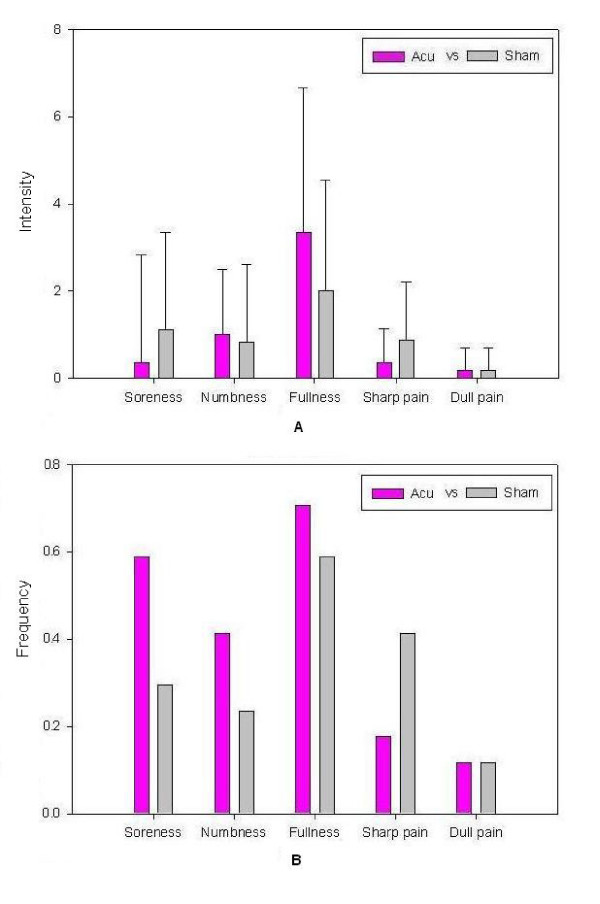
**Psychophysical ratings**. A: The averaged index of subjective pain experienced by all participants (n = 18); only five sensations were listed with mean scale above zero (meaning that at least one subject experienced this sensation). Significant difference of verum acupuncture stimulation compared to the sham stimulation condition was observed in soreness, fullness and sharp pain level. Error bars were based on 95% Confidence Interval. B: The frequency of the sensations occurred among all the subjects. Error bars were based on 95% Confidence Interval. Although the sensations such as soreness, numbness and fullness occurred at higher rates among the subjects under acupuncture intervention as compared to sham stimulation, no significant difference in the overall frequency of experience was found between acupuncture and sham conditions using Fisher's test (*P *> 0.05).

At the end of each verum or sham run, the participant was questioned about aching, pressure, soreness, heaviness, fullness, warmth, coolness, numbness, tingling, dull or sharp pain and any other sensations felt during the stimulation. The intensity of each sensation was measured on a scale from 0 to 10 (0 = no sensation, 1–3 = mild, 4–6 = moderate, 7–8 = strong, 9 = severe and 10 = unbearable sensation). The participants signaled their feelings by raising one finger if the needling sensation became very strong (the subjective rating score ≥ 8), and raising 2 fingers for experiencing sharp pain. The acupuncturist would then adjust the intensity of stimulation by reducing the rotation angle of the needle. In general, the discomfort would disappear almost immediately [2]. Subjects were excluded from functional connectivity analysis if they experienced sharp pain (greater than the mean by more than two standard deviations). The psychophysical data obtained after ACUP or SHAM runs were summarized in Figure 2. Among eighteen participants, only one was excluded from further analysis due to remarkable sharp pain.

### Image pre-processing

For BLOCK and REST runs, the data were preprocessed by removing the first 5 time points to eliminate nonequilibrium effects of magnetization. For both ACUP and SHAM runs, only the datasets after manipulation were selected (total of 500 time points, the same time points as in REST run), and the first 5 time points were discarded in order to obtain a stable resting state. The remaining time points (labeled by blue-color in Fig. 1) were used for functional connectivity analyses.

All functional images were motion-corrected by using a new arithmetic model proposed by Freire et al. [25] in order to reduce global correlation. This method can effectively attenuate the contribution of the global movement to the correlation coefficient. In this work, we used a medical pulse oximeter to monitor the cardiac component, and thereby obtained its spectrum in the low-frequency band. Through a down-sampling process, physiological noise sources and significant artifacts could be removed. The translation and rotation were checked, and the images with head movement greater than 1 mm in any direction or head rotation greater than one degree were excluded. The data were further processed with spatial normalization in the MNI space and re-sampled at 2 × 2 × 2 mm^3 ^using SPM2. Global means and linear trends were removed to eliminate both global correlation and gross signal drifts using Gramm-Schmitt orthogonalization [26]. The datasets were spatially smoothed with a 6 mm FWHM Gaussian kernel.

The low-frequency components of fMRI time series have been shown to have interregional correlations between functionally related brain areas [27]. A finite-impulse response band-pass filter was applied to the dataset used for functional connectivity analyses in order to remove the frequency out of the 0.01–0.1 Hz signals.

### Regions of interest

**Figure 3:**
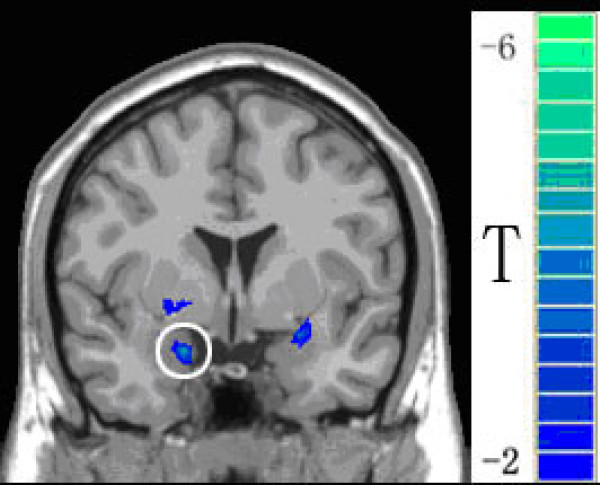
**Functional activation map determined by group analysis in a conventional fMRI study using BLOCK design**. Only negative activation (indicated by *t *values) in the BLOCK run was shown for localizing an ROI in the left amygdala (the blue circle). This ROI was used as a seeding area for ensuing connectivity analysis.

Data from the BLOCK run were used to localize ROIs for further functional connectivity analysis. For this purpose, a four-step process was undertaken. (i) Statistical analysis was performed on individual data by cross-correlating the temporally smoothed boxcar reference function with the time courses of each voxel [28], hence the individual r-map was obtained. (ii) In order to eliminate variance for each condition of interest across subjects, a random-effect analysis was performed with a one-sample t-test (df = 16) at each voxel across subjects based on their individual r-maps [29]. Voxels with |t| > 3.5 (P < 0.001, uncorrected) and clusters with a threshold size > 6 voxels were then superimposed on a high-resolution anatomical image (Fig. 3). (iii) The peak voxel in the brain region (in our case, it is the voxel with the largest negative t value in the left amygdala was obtained). The peak voxel and its 6 nearest neighbors were defined as the ROI. (iv) Due to anatomical variance across the subjects, the subject-specific peak voxel and subject-specific ROI were defined on individual r-maps as follows: the ROI from the above group analysis was taken as a mask and then, based on individual r-maps, the voxel with the largest r-value within this mask was taken as the subject-specific peak voxel. Regarding the subject variance and improving the ratio of signal to noise, this voxel together with its 4 nearest neighbors were used as a subject-specific ROI. The averaged time courses of voxels within the ROI were used as a single low-frequency reference function named as the 'seeding' time course.

### Functional connectivity analysis

For each subject, the 'seeding' time courses of both contralateral amygdala were respectively cross-correlated with all low-pass filtered voxels to generate functional connectivity maps within each of the three conditions. Since the brain responses during the block run (Table 1) were found similar to the previous study [2], only the left amygdala was used in our further connectivity analyses of the related brain network. This approach was termed as within-condition interregional covariance analysis (WICA) [14]. The resulting correlation coefficient *r*-maps were normalized and corrected to roughly standard normal distributions using the methods previously described [30,31]. The normality of the distribution was then tested using Kurtosis tests (P < 0.05). The three z-maps of each individual were entered into one-sample t-tests respectively [29] to determine whether the group data was significantly different from zero.

**Table 1:** The main results of group analysis of block data.

**Anatomical regions**	Block					
	
			**Talairach**			
	Hem	BA	x	y	z	t
**Amygdala**	R		21	-4	-13	-6.33
	L		-24	-6	-12	-6.18

**Hippocampus**	R		30	-19	-10	-5.01
	L		-22	-18	-21	-5.32

**Hypothalamus**	R		3	-1	9	-6.15
	L		-1	-4	16	-7.38

**ACS**	R	32	5	29	-5	-6.21
	L	24	-2	27	-3	-6.39

**ACP**	R	24	2	29	4	-5.36
	L	24	-5	31	3	-5.02

**PMC**	R	24	2	-18	31	-4.08
	L	24	-9	-15	35	-4.38

**Posterior cingulate**	R	23	5	-38	31	5.01
	L	23	-3	-31	30	5.12

**Anterior insula**	R		40	10	9	4.92

**Thalamus**	R		18	-26	6	6.98
	L		-13	-33	4	6.32

**SII**	R		62	-7	21	5.81
	L		-60	-3	28	5.62

(*df *= 16, *P *< 0.001, uncorrected)

ACS, Anterior cingulate-subgenual; ACP, Anterior cingulate-pregenual; PMC, Posterior middle cingulated; Abbreviations: BA-Brodmann Area; Hem -Hemisphere.

In order to quantitatively compare the functional connectivity among these three conditions (i.e. REST, ACUP, SHAM), the strength of a connection between two brain regions was calculated by weighing the t-values and volumes on the basis of the averaged cross-correlations with the 'seed' and then normalized [14]. Furthermore, the paired t-tests were applied on a voxel-by-voxel basis over all the subjects to contrast the functional connectivity maps (i.e. ACUP – REST or SHAM – REST). The difference map was used to reveal how acupuncture or sham stimulation may modulate the resting-state functional connectivity. Similarly, paired t-tests were used to contrast between ACUP and SHAM conditions for showing an acupuncture specific modulation (i.e. ACUP – SHAM). All the resulting t-maps were then cluster-filtered to remove correlations involving less than 3 contiguous voxels and then superimposed on high-resolution anatomical images using a P < 0.01 cutoff threshold (FDR corrected). The above image processing programs were coded in MATLAB7 (The MathWorks, Inc.).

## Results

**Figure 4:**
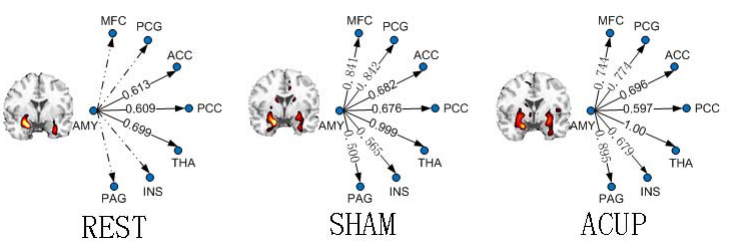
**Amygdala-associated functional brain networks**. A: the resting condition (REST); B: the post-sham condition (SHAM); C: the post-acupuncture condition (ACUP). The functional connectivity maps on the left show the statistical significance level of the correlation (t > 5.8, df = 16, P < 0.01 FDR corrected) in a coronal brain section through the amygdala. The amygdala-associated networks on the right show the strength of a functional connection (the weighted and normalized correlations) between the left amygdala and other selected brain regions. Dotted lines indicate statistically insignificant correlations. For abbreviations refer to Table 1.

In the BLOCK experiment, acupuncture stimulation induced fMRI-BOLD signal changes over extensive brain areas such as the hippocampus, hypothalamus, ACC, posterior cingulate cortex (PCC), anterior insula, thalamus, and SII. These results are consistent with previous fMRI studies, especially well-defined deactivation in the left amygdala (Fig. 3) [2]. Based on the activation study, three brain networks were defined in the ensuing connectivity analyses using the activated left amygdala as a reference (Table 2 and Fig. 4). We found an amygdala-associated brain network, consisting of extensive areas in the frontal gyrus, temporal gyrus, ACC, PCC, thalamus and basal ganglia (Table 2). Besides showing the overlapped regions with the above network, the post-acupuncture condition engaged other brain regions including the medial prefrontal cortex (MPC), postcentral gyrus (PCG), insula, and PAG. In addition, the post-sham network covers more areas (reflected in the number of voxels) in the cingulate and basal ganglia, and has stronger between-region correlations (reflected in the t values). While the post-acupuncture condition overlapped extensive regions with the post-sham network, there were changes in the connectivity pattern with respect to both the size and strength of the localized correlations in the inferior temporal gyrus (ITC), PCG, cingulate, insula and PAG (Table 2 and Fig. 4).

**Table 2:** Functional localization of the brain regions showing significant correlations with the ROI in the left amygdala (*df *= 16, *P *< 0.01, FDR corrected).

	ACUP							SHAM						REST					
	
**Anatomical regions**			Talairach						Talairach						Talairach				
	Hem	BA	x	y	z	*t*	Vol	BA	x	y	z	*t*	Vol	BA	x	y	z	*t*	Vol
		
**Medial Prefrontal**	L	10	-6	51	6	5.56	270	11	-11	49	-16	9.51	189						
**Cortex (MFC)**	R							6	6	0	51	5.05	81						
							
**Postcentral Gyrus**	L	3/40	-30	-36	57	5.59	378	1/3	-42	-21	60	5.72	810						
		
**Temporal Gyrus**	L	20/37	-51	-39	-21	6.28	1242	20	-54	-48	-18	7.20	1377	22	-51	-60	12	4.50	1593
	R	20/37	51	-39	-21	6.24	1620	20	39	-12	-33	5.71	189	20	51	-54	-18	4.89	621
		
**Anterior Cingulate**	L													25	-6	-23	-10	4.01	81
**Cortex (ACC)**	R	32	9	36	-9	6.01	162	32/33	6	21	24	5.42	135	32	10	41	-3	4.21	81
		
**Posterior Cingulate**	L	29	-9	-45	3	5.12	54	30/31	-12	-63	9	5.66	216	30	-12	-63	9	4.64	54
**Cortex (PCC)**	R							30/31	15	-54	3	5.38	54	30	15	-54	3	4.68	81
		
**Thalamus (THA)**	L		-3	-18	0	6.58	1647		-3	-12	2	5.60	1674		-9	-21	0	4.51	162
	R		6	-18	0	6.30	2133		6	-9	9	6.76	3483						
							
**Insula (INS)**	R	13	39	3	9	5.23	135	13	39	9	-6	4.9	54						
		
**Putamen**	L		-27	3	0	6.87	3348		-27	-6	-6	6.69	2918		-21	0	-6	5.48	891
	R		21	3	-3	6.03	1620		27	-3	-6	6.05	2170		-24	0	-6	5.32	837
		
**Caudate**	L		-9	15	3	5.92	1836		-15	6	18	6.01	1890		-12	18	-6	4.75	243
	R		6	12	-6	5.81	162												
		
**Periaqueductal Gray**	L		-4	-29	-12	6.18	2268												
**(PAG)**	R		2	-22	-9	5.46	567		+4	-25	-8	4.11	54						

Abbreviations: BA-Brodmann Area; Hem -Hemisphere; Vol-Volume (mm^2^)

For a quantitative presentation of the connectivity changes over different conditions, eight ROIs including the amygdala, were defined in Figure 4 to construct a basic connectivity module [14]. We selected these eight regions by considering that (1) they were activated in our BLOCK experiment during acupuncture stimulation and (2) they have been implicated in both sensation and affective pain perception, as well in the pain-modulation system [2,32,33]. While the regions in this brain module were active during or even following acupuncture stimulation, the connections among these regions may or may not be expressed (as shown by the statistical significance level of the correlations in Fig. 4). Furthermore, the between-region correlations changed over different conditions. For instance, while the connections between the amygdala and cingulate remained almost the same, all the other selected connections were significantly increased during SHAM relative to REST, suggesting modulation of the resting network by sham stimulation. Most importantly, the increases in connectivity with the amygdala during ACUP (relative to SHAM) were found specifically in the PAG and INS. While functional connections to the ACC and thalamus remained the same between SHAM and ACUP, the connections to the MFC, PCG (the SII) and PCC were decreased. Note that the connectivity maps covered the contralateral amygdala and it also showed a different degree of correlation with the reference under each condition. For simplicity, the results in the cerebellum were not shown in the above analysis.

**Figure 5:**
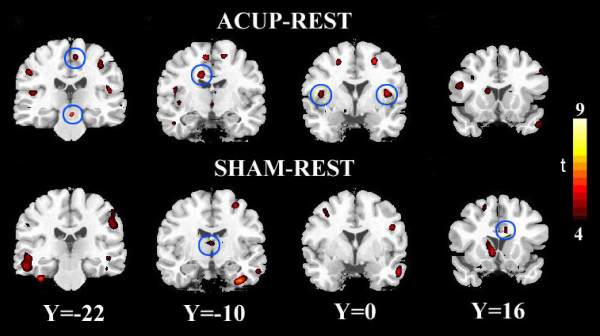
**Changes in the resting-state functional network following verum acupuncture or sham stimulation**. Difference maps were obtained by comparisons between the connectivity maps (ACUP-REST or SHAM-REST) using paired t-tests with a threshold of t > 4.0 (df = 16, P < 0.01, FDR corrected). The color-coded maps indicate brain regions that have significant increases in functional connectivity relative to the resting-state.

Figure 5 and Table 3 showed the contrast of connectivity maps between the two conditions by voxel-wise t-tests, further indicating the brain regions in which the REST connectivity was modulated by the ACUP or SHAM condition. Both verum and sham induced significant changes in the resting-state functional connectivity. Moreover, while the locations of these connectivity changes had extensive overlap between verum and sham there were apparent differences between post-acupuncture modulation and post-sham modulation (Fig. 5 and Table 3).

**Table 3:** Localization of connectivity maps by comparing between the ACUP vs. Rest, and SHAM vs. Rest using paired *t*-tests (*df *= 16, P < 0.01, FDR corrected).

	Acu-Rest							SHAM-Rest					
		
**Anatomy regions**			**Talaiarch**						**Talaiarch**				
	Hem	BA	x	y	z	t	Vol	BA	x	y	z	t	Vol
	
**MFC**	L	11	-4	24	-12	4.98	108	11	-10	43	-11	5.67	274
	R							11	9	48	-12	5.32	263
	
**ACC**	L	24/32	-9	41	6	4.88	98	24	-3	3	29	4.08	28
	R	24/32	8	40	2	4.93	126						
	
**PCC**	L							26/23	-4	-38	21	4.64	116
	R							26/23	10	-47	25	4.30	98
	
**Insula**	L	13	-36	2	8	5.72	243	13	-39	-11	12	4.98	118
	R	13	44	2	12	5.49	261	13	32	16	5	4.61	92
	
**ITC**	L	20	-50	-10	-24	5.01	232	20	-40	-10	-26	6.39	318
	R	20	43	-10	-30	5.12	118	20	45	-10	-36	6.21	432
	
**MTC**	L	21	-62	-20	12	5.21	124	21	62	-18	-12	6.92	833
	R	21	59	-50	-12	6.38	612	21	-61	20	11	5.12	204
	
**Caudate**	L		-12	16	12	5.83	1182		-7	16	-8	6.32	1614
	R		16	14	11	5.71	136		8	17	11	6.12	324
	
**Thalamus**	L		-6	-11	4	5.97	646		-4	-10	10	6.02	812
	R		8	-16	2	5.88	312		6	-10	5	5.92	489
	
**Precentral**	L	4	-7	-22	52	5.62	204	4/6	-35	-21	63	5.56	215
	R							4/6	29	-22	65	5.41	183
	
**Postcentral**	L	3/4	-37	-22	54	6.31	513	3/4	-47	-24	51	6.73	1008
	R	3/4	39	-22	50	6.19	465	3/4	50	-22	40	6.24	842
	
**PAG**			5	-28	-11	4.91	102						

Positive *t *values indicate ACUP or SHAM > Rest.

Abbreviations: ITC-Inferior Temporal Cortex; MTC-Middle Temporal Cortex.

**Figure 6:**
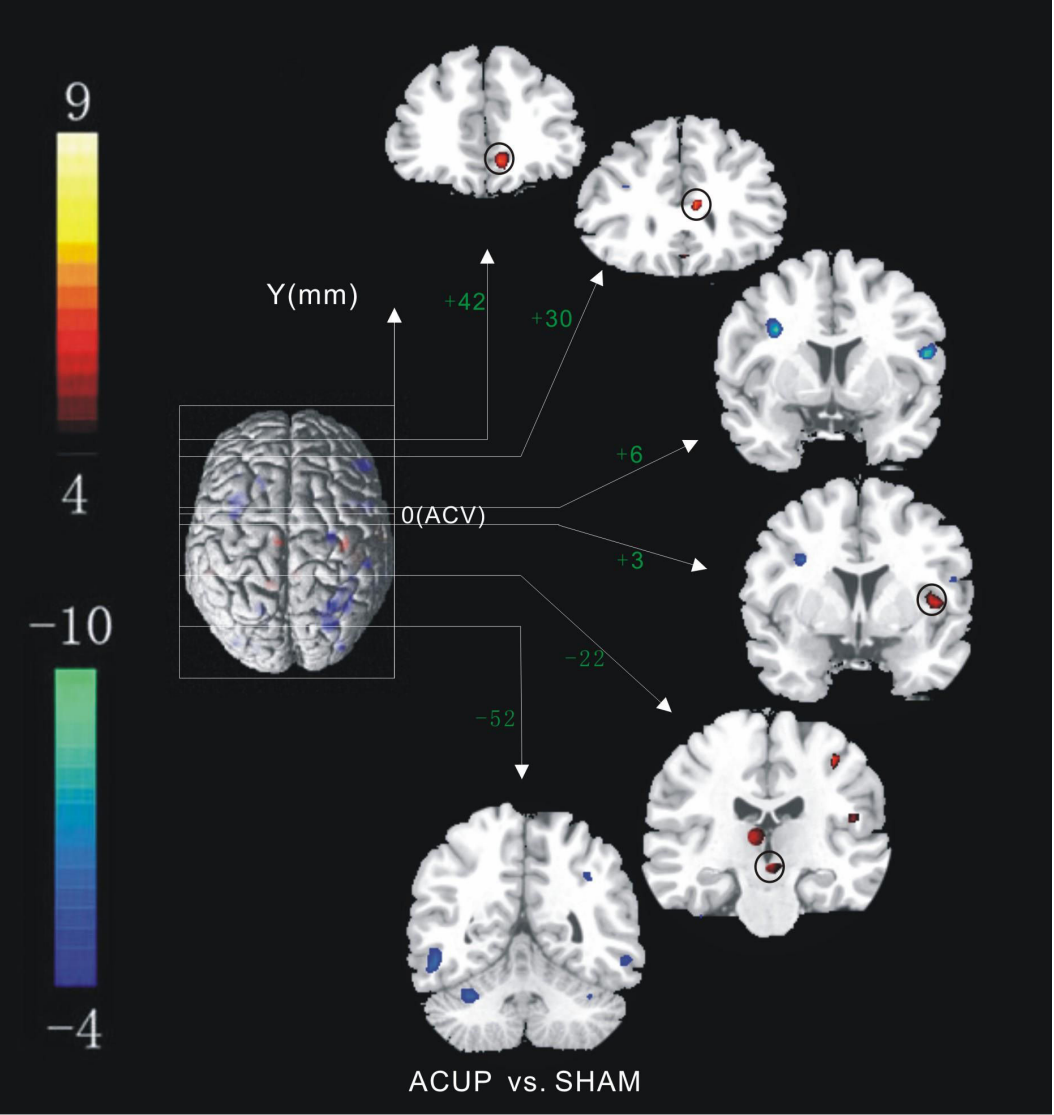
**Paired t test between post-acupuncture connectivity map and post-sham connectivity map**. The significance threshold was t > 4.0, df = 16, p < 0.01 FDR correct. The red regions with positive t values indicate ACUP > SHAM, and the blue regions with negative t values indicate SHAM > ACUP. y (mm): Talairach coordinates indicate the locations of the coronal brain sections (ACV: anterior commissure verticalization).

To further demonstrate acupuncture-specific effects on the modulation of resting-state connectivity, we directly compared the SHAM and ACUP connectivity maps using voxel-by-voxel paired t-tests (Fig. 6 and Table 4). Our results showed that the SI, SII, ITC and cerebellum were more associated with the amygdala during sham relative to verum acupuncture; these regions constructed a post-sham network. In contrast, the ACUP-induced increases in the amygdala connectivity were primarily found in the PAG and insula, which constructed a post-acupuncture network.

**Table 4:** Localization of acupuncture specific effects by comparing between the ACUP and SHAM connectivity maps using paired *t*-tests (*t *= 4.0, *df *= 16, P < 0.01, FDR corrected).

	**ACUP – SHAM**						
	
			**Talairach**				
**Anatomy regions**	Hem	BA	x	y	z	t	Vol
Medial Frontal Gyrus (MFG)			9	39	-9		

Precentral Gyrus	L	6	-33	3	27	-6.28	270
	R	4	36	-21	54	5.93	54

Inferior Frontal Gyrus (IFG)	R	44	54	9	15	-5.75	216
						
Postcentral Gyrus (SII)	R	2	54	-30	57	-5.68	54
						
Superior Parietal Gyrus	R	7	27	-57	48	-5.43	81

Inferior Temporal Gyrus (ITG)	L	20	-42	-21	-33	-6.50	216
	R	21	39	-6	-36	-5.84	27

Insula (INS)	L	13	-33	-3	18	4.88	108
	R	13	45	-27	18	5.33	216

Anterior Cingulate Cortex (ACC)	L	24/32	-6	30	12	5.11	81
	R	24/32	9	36	-9	5.46	270

Uncus	R	20	36	-12	-36	-5.77	81
						
Caudate	R		12	15	12	-6.27	108
						
Putamen	R		24	12	9	-5.11	54

Periaqueductal Gray (PAG)			0	-22	-8	5.28	182

Cerebellar Culmen	L		-36	-54	-27	-6.73	216
	R		39	-54	-27	-5.75	189

Cerebellar Tonsil	L		-27	-57	-48	-6.83	216
						
Cerebellar Declive	L		-39	-60	-21	-6.02	513
	R		36	-57	-27	-6.63	108
						
Cerebellar Pyramis	L		-18	-72	-36	-5.55	108
						
Cerebellar Tuber	L		-54	-60	-33	-5.7	54

Positive *t *values indicate ACUP > SHAM, while negative *t *values indicate SHAM > ACUP.

Abbreviations: BA-Brodmann Area; Hem – Hemisphere; Vol-Volume (mm^3^).

**Figure 7:**
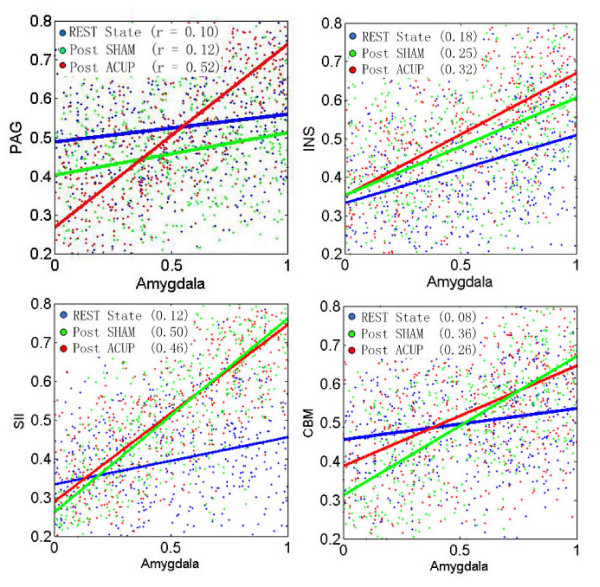
**Regression results of amygdale and other representive brain regions**. Dynamic changes in the interaction between the amygdala and other representative brain regions implicated in pain sensation and pain modulation over different states: REST, Post-SHAM, and Post-ACUP. Regression analyses were based on normalized BOLD signal intensities calculated individually from the time courses of the ROIs: the amygdala, PAG, Insula (INS), SII and cerebellum (CBM). The resulting regression slopes (r) were averaged over 17 subjects (for clarification, the scattered data points shown are from only one subject.

Based on the selected ROIs that were implicated in both verum and sham networks, we performed a regression analysis to characterize how the dynamic changes in these specific brain regions interacted with the amygdala activities (Fig. 7). The slopes in the PAG and insula were apparently steeper during verum than those during sham acupuncture (the upper two panels in Fig. 7), which are consistent with the above results that the PAG and insula are more strongly involved in specific acupuncture effects. On the other hand, the slopes in the SII and cerebellum are steeper during SHAM than those during verum acupuncture (the lower two panels in Fig. 7), indicating that these two structures are more strongly involved more in the sham response.

## Discussion

In this paper, we employed functional connectivity analysis methods with a new NRER-fMRI design to investigate the sustained effects of acupuncture. In most previous fMRI studies, the model-dependent analysis methods using a block design require prior knowledge of event timing from which an anticipated hemodynamic response can be modeled. However, this type of analysis methods cannot be used without a predictable hemodynamic response reflecting the actual BOLD signal changes induced by acupuncture. In the current study, we used a model-independent approach, i.e. the new NRER-fMRI design, to explore systematic changes of the BOLD signal without a decrease in the statistical power and the bias on the results.

Our work presents the first fMRI connectivity study of acupuncture using a modular approach in connectivity analysis [14], followed by a more recent connectivity study on the defaut mode network [33]. We have defined a resting-state brain network that is associated with an amygdalar region activated during acupuncture stimulation (Fig. 4). Our results further showed that the amygdalar-specific network consisting of brain regions overlapped with the pain-matrix to some extent. These regions include extensive areas in the SI and SII, insula, ACC and PFC as well as the hypothalamus and PAG (Table 1). While these brain regions were activated by sham or acupuncture stimulation, the corresponding network associated with sham could be dissociated from acupuncture modulation effects by differentiating functional connectivity patterns among these regions (Fig. 4).

As the amygdala plays a dual role of facilitating as well inhibiting in the modulation of pain behavior and nociceptive processing at different levels of the pain matrix, the amygdala-associated network during the resting state may be crucial in both pain and analgesia systems implicated in the effects of acupuncture stimulation [2,20,34]. Many previous studies have found a basic network derived from the important concept of "default mode" in the resting brain [10,35]. Within this default mode, the temporal correlation of the fMRI signal during a resting-state provides complementary information about the intrinsic interaction between different brain regions. With such a baseline, the verum acupuncture- or sham-induced changes in the temporal correlation may represent the modulation of region-to-region interactions. It should be noted that the low-frequency temporal correlations in the resting state are also related to uncontrolled brain activities [36]. Continuous stimulation during ACUP or SHAM may result in a higher degree of low frequency correlation than the "resting" uncontrolled stimulus state in these regions, which may provide an explanation for the increase in connectivity in both sham and acupuncture conditions (Figs. 4, 5).

The change in the region-to-region connectivity is an indication of functional changes of the network, and may provide complementary information for exploring modulating effects of acupuncture or sham on the network of interest (Figs. 4, 5). Based on the pain-related network in the resting brain, we can expect that either verum or sham acupuncture (using pain-related stimulation) may modulate the functional connectivity in some specific brain regions implicated in this network. The connectivity network during the post-acupuncture condition was similar to the sham except for more extensive and stronger connectivity in the limbic system. Furthermore, since the procedure during SHAM intervention, as a whole, is the similar to that of ACUP and all of subjects are naïve to acupuncture, we expect that SHAM is believed to be the same procedure as verum acupuncture to our subjects in terms of potential placebo effects of pain relief. Therefore, the observed differences between these two conditions may constitute a specific physiological effect. Comparing ACUP and SHAM, we found connectivity increases specifically in the PAG and INS (Fig. 4). The PAG has abundant opiate receptors and participates in both opioid analgesia and acupuncture analgesia [5,8]. In addition, a recent PET study involving patients in pain, has clearly identified a hyperactivation of the ipsilateral insula, suggesting a specific neural structure underpinning the effect of acupuncture for the treatment of chronic musculoskeletal pain. The insula is a key modulator of the visceromotor system. The increases in the connectivity of the amygdala with both the insula as well PAG indicate that the uncrossed visceroceptive autonomic pathways may be engaged, which seems to be crucial for acupuncture analgesia effects reflecting its specific action on the central nervous system.

It is now believed that sham does have a physiological effect, as well as many of the central neural substrates may be involved in the sham-related pain sensation. Compared to the ACUP, an increased connectivity was shown among the MFC, PCG (and the SII) and PCC during the SHAM. The MFC has been shown to be involved in the modulation of pain by regulating attention and affective emotion [8,9]. The MFC and PCC may also transform the memory or sensory information to assign meaning to pain, and subserve planning and execution of coping strategies. Previous imaging studies have shown that the SII and PCC have connections to memory-related temporal-lobe structures and the motor system, suggesting that these cortices may contribute to learning and memory of pain, as well as to pain-motor integration [37-40]. In addition, the PCC near the 'unpleasantness region' is associated with response selection, conflict monitoring and attention, which is considered to be more reliably activated by pain [37-39]. Therefore, the increased connections with the amygdala shown in the SII and PCC during SHAM may be due to more intense sensations commonly induced by sham stimulation. As a result, we speculate that the action of sham may involve non-specific effects supporting both sensory and affective pain perception. Although no significant statistical differences between acupuncture and sham scores on subjects' perceptions of sensations (P < 0.05), the sharp pain levels showed an elevated tendency during the sham stimulation (paired t-test, P < 0.07), primarily due to the low scores (typically less than 1) measured during the acupuncture stimulation.

The involvement of the ACC in both sham and acupuncture is an interesting finding in our study. The functional connectivity results are consistent with the previous neuroanatomical and electrophysiological findings that the efferents of the amygdala have bidirectional relations with the ACC [41-43]. Although a considerable volume of literature documented the role of the ACC in autonomic regulation and emotion [44], some studies pointed out that a pain response as shown in the ACC might be associated with the "suffering" component of pain [45] or with the opioid pathway [46,47]. Placebo effects following conditioning with surreptitious variation of heat pain can induce decreased activity within the ACC. We hypothesized that placebo analgesia may arise from changes in the expectation of pain within higher cognitive centers such as the ACC. The ACC contains a high concentration of opioid receptors [48], and has been regarded as a functional region in opioid analgesia and in other forms of pain modulation [49,50], which may suggest a similar involvement of higher order control of opioid-dependent placebo analgesia. We therefore suggest that the ACC activation found in both ACUP and SHAM is linked to the expectation of therapeutic benefit and exerts a top-down effect on the midbrain which is at the same site of the PAG activation reported by Petrovic et al [51]. Previous studies also showed that acupuncture might stimulate both pain modulation and analgesia systems by releasing endogenous opioids [52]. However, there was no significant difference in the amygdala-ACC connectivity between SHAM and ACUP (Fig. 4.), suggesting that the ACC may not mediate the effects specific to the sustained effects of acupuncture, but participate in non-specific components such as expectation and pain-related affective processes.

The activation of the insula has been reported in previous acupuncture studies [9,53,54]. The increased connectivity of the insula in our study is consistent with electrophysiological studies and clinical investigations [55], which showed the insula's involvement in emotional processing (fear, uneasiness, etc) and ascending visceral symptoms [56]. The involvement of the insula in the post-acupuncture functional network is also consistent with TCM's viewpoint that the known healing effects of Zusanli (ST36) acupuncture on gastroenteric disorders such as gastroenteritis and gastroenteric spasm may be mediated through the insular visceral feedback pathway. Furthermore, the unaltered connectivity of the ACC may therefore suggest that the expectation during the treatment may have a physiological effect on the brain, which mediates a potentially powerful non-specific response to acupuncture. On the other hand, our results showed that the SII and cerebellum were more associated with SHAM, suggesting that the post-sham effects may mostly be represented in modulating responses in sensory processes [2]. More compelling evidence supporting central effects specific to acupuncture was from direct comparisons between the ACUP and SHAM connectivity maps (Fig. 6 and Table 4). Our ROI-based temporal analyses in Figure 7 indicated a dynamic relationship between the amygdala and the other four regions implicated in the pain-related network. When the region-to-region connectivity patterns were further explored, our results showed differential modulatory effects of acupuncture and sham stimulations on the corresponding networks, in which the modulatory effects were mediated in a time-dependent way. Therefore, these findings suggest that the difference in functional connectivity is region-specific, which provides indirect evidence in support of the discrepancy between verum and sham acupuncture.

It has been recently suggested that acupuncture may be effective in pain relief regardless of acupoint locations, although there are differences in their efficacies [57]. However, as shown in our results using a new fMRI approach, the verum acupuncture and sham stimulation (at a non-acupoint) induced the activations of differential brain networks. The specific pattern of correlation during the post-acupuncture condition provides a reasonable explanation for the actual analgesia effect of acupuncture as well as direct evidence supporting that an acupuncture point may have its own functional specificity.

## Conclusion

Using connectivity analysis with the new NRER-fMRI design, we demonstrated that there is an amygdala-associated resting brain network, which can be further modulated by sham and acupuncture stimulations, and that the specific effects of acupuncture may result from the cooperation of brain regions engaged in the resting functional network. In addition, this network encompasses the brain structures that are implicated in both pain sensation and pain modulation.

## Competing interests

The authors declare that they have no competing interests.

## Authors' contributions

WQ carried out the experiment and wrote the manuscript. JT participated in the design and coordination of this study. LB participated in the data processing and provided assistance in writing the manuscript. XP participated in the design of this study. PC performed the entire acupuncture procedure. JD participated in the design and coordination of this study. KMvD provided assistance in writing and revising the manuscript. YL provided fMRI methodology in the study and assisted in writing the manuscript. All authors read and approved the final manuscript.
